# Interactive Two-Way mHealth Interventions for Improving Medication Adherence: An Evaluation Using The Behaviour Change Wheel Framework

**DOI:** 10.2196/mhealth.9187

**Published:** 2018-04-12

**Authors:** Nicole Chiang, Michael Guo, K Rivet Amico, Lou Atkins, Richard T Lester

**Affiliations:** ^1^ Independent Researcher Vancouver, BC Canada; ^2^ Faculty of Medicine University of British Columbia Vancouver, BC Canada; ^3^ Research Associate Professor Department of Health Behavior and Health Education University of Michigan Ann Arbor, MI United States; ^4^ Centre for Behaviour Change University College London London United Kingdom; ^5^ Division of Infectious Diseases Department of Medicine University of British Columbia Vancouver, BC Canada

**Keywords:** mHealth, chronic disease, medication adherence, primary health care, disease management

## Abstract

**Background:**

Medication adherence is an important but highly complex set of behaviors, which for life-threatening and infectious diseases such as HIV carry critical consequences for individual and public health. There is growing evidence that mobile phone text messaging interventions (mHealth) connecting providers with patients positively impact medication adherence, particularly *two-way* engagement platforms that require bidirectional communication versus *one-way* in which responses are not mandatory. However, mechanisms of action have not been well defined. The Behavior Change Wheel is a comprehensive framework for behavior change that includes an all-encompassing model of behavior known as Capability Opportunity Motivation-Behavior and is complemented by a taxonomy of behavior change techniques. Evaluating mHealth interventions for medication adherence using these tools could provide useful insights that may contribute to optimizing their integration into the healthcare system and successful scaling-up.

**Objective:**

This study aimed to help address the current knowledge gap regarding how two-way mHealth interventions for medication adherence may work by applying the Behavior Change Wheel to characterize WelTel: an interactive digital health outreach platform with robust evidence for improving adherence to antiretroviral therapy.

**Methods:**

To characterize how WelTel may promote medication adherence, we applied the Behavior Change Wheel to systematically (1) generate a *behavioral diagnosis* through mapping known antiretroviral therapy adherence barriers onto the Capability Opportunity Motivation-Behavior model of behavior, (2) specify the behavior change techniques that WelTel delivers, (3) link identified behavior change techniques to corresponding intervention functions of the Behavior Change Wheel, and (4) connect these behavior change techniques and intervention functions to respective Capability Opportunity Motivation-Behavior influences on behavior to determine potential mechanisms of action.

**Results:**

Our evaluation of WelTel using the Behavior Change Wheel suggests that most of its impact is delivered primarily through its personalized communication component, in which 8 different behavior change techniques were identified and linked with 5 intervention functions (environmental restructuring, enablement, education, persuasion, and training). Its mechanisms of action in promoting antiretroviral therapy adherence may involve addressing all Capability Opportunity Motivation-Behavior influences on behavior (physical and psychological capability, physical and social opportunity, reflective and automatic motivation).

**Conclusions:**

Systematically unpacking the potential active ingredients of effective interventions facilitates the creation and implementation of more parsimonious, tailored, and targeted approaches. Evaluating WelTel using the Behavior Change Wheel has provided valuable insights into how and why such interactive two-way mHealth interventions may produce greater impact than one-way in addressing both nonintentional and intentional forms of nonadherence. The application of the Behavior Change Wheel for evidence synthesis across mHealth interventions targeting various conditions would contribute to strengthening the knowledge base regarding how they may work to impact medication adherence behavior.

## Introduction

### Medication Adherence Is a Significant Global Challenge

In chronic disease management worldwide, only 50% of patients on average adhere to their prescribed long-term therapies [1]. Medication adherence is an important, but highly complex set of behaviors, which for life-threatening and infectious diseases such as HIV carry critical consequences for individual and public health. Adherence to antiretroviral therapy (ART) for people living with HIV combats chronic infection through viral suppression, optimizes an individual’s immune recovery, mitigates drug resistance, and reduces odds of viral transmission [2-4]. Viral suppression achieved through adherence is essential for achieving global “90-90-90” targets declared by the Joint United Nations Programme on HIV/AIDS: by 2020, 90% of all people living with HIV will know their status, 90% of all people diagnosed as HIV-positive will receive uninterrupted ART, and 90% of all people taking ART will achieve viral suppression [5]. However, suboptimal ART adherence is not uncommon, and realizing the full benefits of ART requires considerable effort for many [6]. Concerted efforts over the past two decades have been geared toward specifying effective interventions for ART adherence globally [6,7]; however, efforts to distil active intervention ingredients remain largely absent. Given that medication adherence is often subject to multilevel influences beyond the individual, investigating mechanisms of action through which interventions may promote this behavior facilitates the delivery of systematically created and targeted approaches. Understanding and specifying the potential underlying mechanisms behind effective interventions is critical for design improvement and successful wide-scale implementation.

### Certain mHealth Interventions may Offer a Solution

Mobile phone interventions for promoting health (mHealth) are becoming increasingly available, and several interventions aim to improve medication adherence across various disease states [8,9]. Among people living with HIV, the World Health Organization recognizes text messaging in their current guidelines as an evidence-based intervention for encouraging ART adherence [10]. However, no distinction has been made between the various types of applications that exist. In particular, *two-way* versus *one-way* engagement platforms are significantly different from each other; the former implies an expectation of patient-provider communication, whereas the latter simply prompts reminders and instructions without mandatory patient feedback. Two-way text messaging is thought to be more effective compared with one-way for inducing ART adherence, and this distinction has important implications on the usage of text messaging for chronic disease management [9,11]. Policy makers in control of health care expenditure may attempt to promote cost saving through spacing clinic visits further apart; therefore, interactive mHealth interventions potentially offer cost-effective and essential touch points between these visits to keep patients engaged in care [12]. In addition, they may prompt the self-management of issues as they occur in a timely manner. Furthermore, such interventions can benefit both patients and health care providers in more resource-limited contexts through addressing demand-side and supply-side challenges that exist in service delivery. Interactive two-way mHealth engagement can help alleviate such health care system strain and improve its efficiency through focusing on patients who are in the most need of care at any given time, while promoting the individualized downstream care pathway that may include tailored patient-provider communication beyond text messaging.

### Ambiguity Exists Today Regarding how mHealth Interventions for Medication Adherence Work

Although there is growing quantitative evidence that two-way text messaging is superior to one-way for improving medication adherence, there remains a significant knowledge gap as to why. Mechanisms of action behind these interventions are currently unspecified. Consequently, this lack of insight may contribute to future challenges with regard to integrating such interventions into health services, and ultimately scaling them up. Attaining strong foundational knowledge of *how* the intervention influences adherence behavior may contribute to maximizing implementation success. In the context of HIV, similar sentiments have been shared in a systematic review focused on unpacking group-based ART adherence interventions to describe how they may work [13].

WelTel is an example of an interactive two-way mHealth intervention that has been shown to improve self-reported 95% ART adherence and viral load suppression rates, as demonstrated by a multisite randomized clinical trial conducted in Kenya [14] and further validated in Canada [15]. Delivery of the digital health outreach platform included a weekly automated text message saying “How are you?” (“Mambo?” in Kiswahili), and the patient was obliged to respond accordingly. If the patient replied “problem” (“Shida” in Kiswahili) or was unresponsive, a care provider initiated a follow-up and personalized care was given.

**Figure 1:**
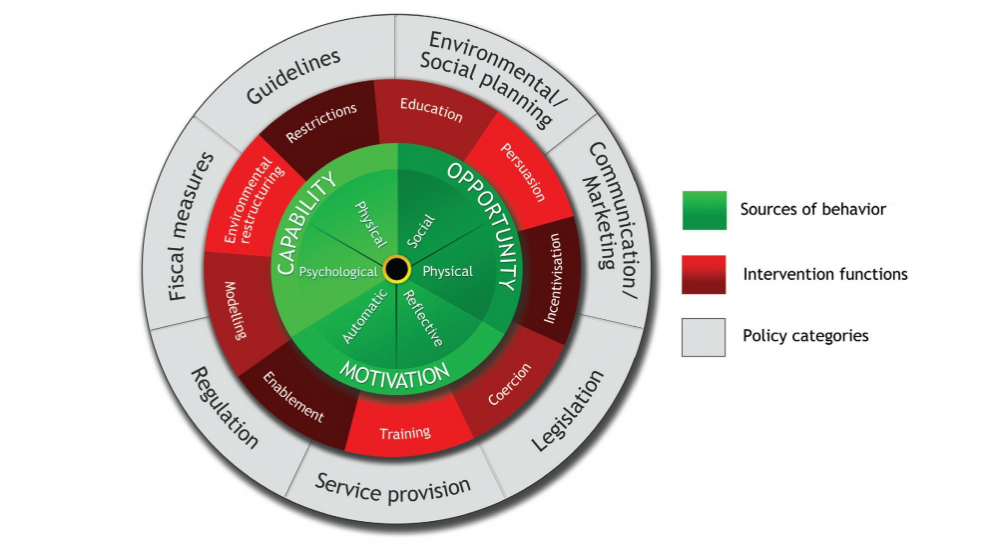
Behavior Change Wheel (image spelling has been modified, based on Michie S et al [18]).

**Figure 2:**
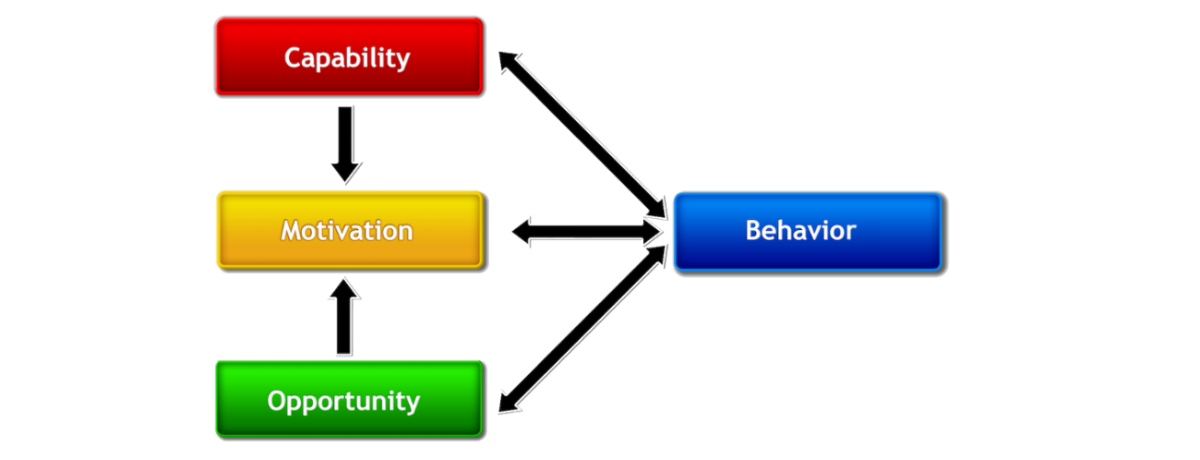
Capability Opportunity Motivation - Behavior (COM-B) model of behavior (image spelling has been modified, based on Michie S et al [18]).

### Objective

The primary objective of this study was to investigate and specify WelTel’s potential mechanisms of action using a comprehensive and evidence-based theory of behavior change framework. The Behavior Change Wheel (BCW) (Figure 1) provides a systematic approach to designing and evaluating interventions, and has been developed through the synthesis of 19 existing behavior change frameworks [16]. It characterizes an array of intervention functions (broad strategies for inducing the target behavior) and policy functions (ways to support and implement these strategies) that can be used within a wide range of contexts. Specifying linkages between intervention functions and influences on the target behavior reveals appropriate strategies for potentially bringing upon change [16]. A complementary behavior change taxonomy further elaborates upon these intervention functions in terms of many specific behavior change techniques (BCTs) [17]. Moreover, the BCW highlights the importance of taking into account multilevel influences on behavior through incorporation of the Capability Opportunity Motivation-Behavior (COM-B) model of behavior (Figure 2).

Using WelTel as a case study, our goal was to help address the current research gap regarding the qualitative evidence of two-way mHealth interventions for improving medication adherence.

## Methods

**Figure 3:**
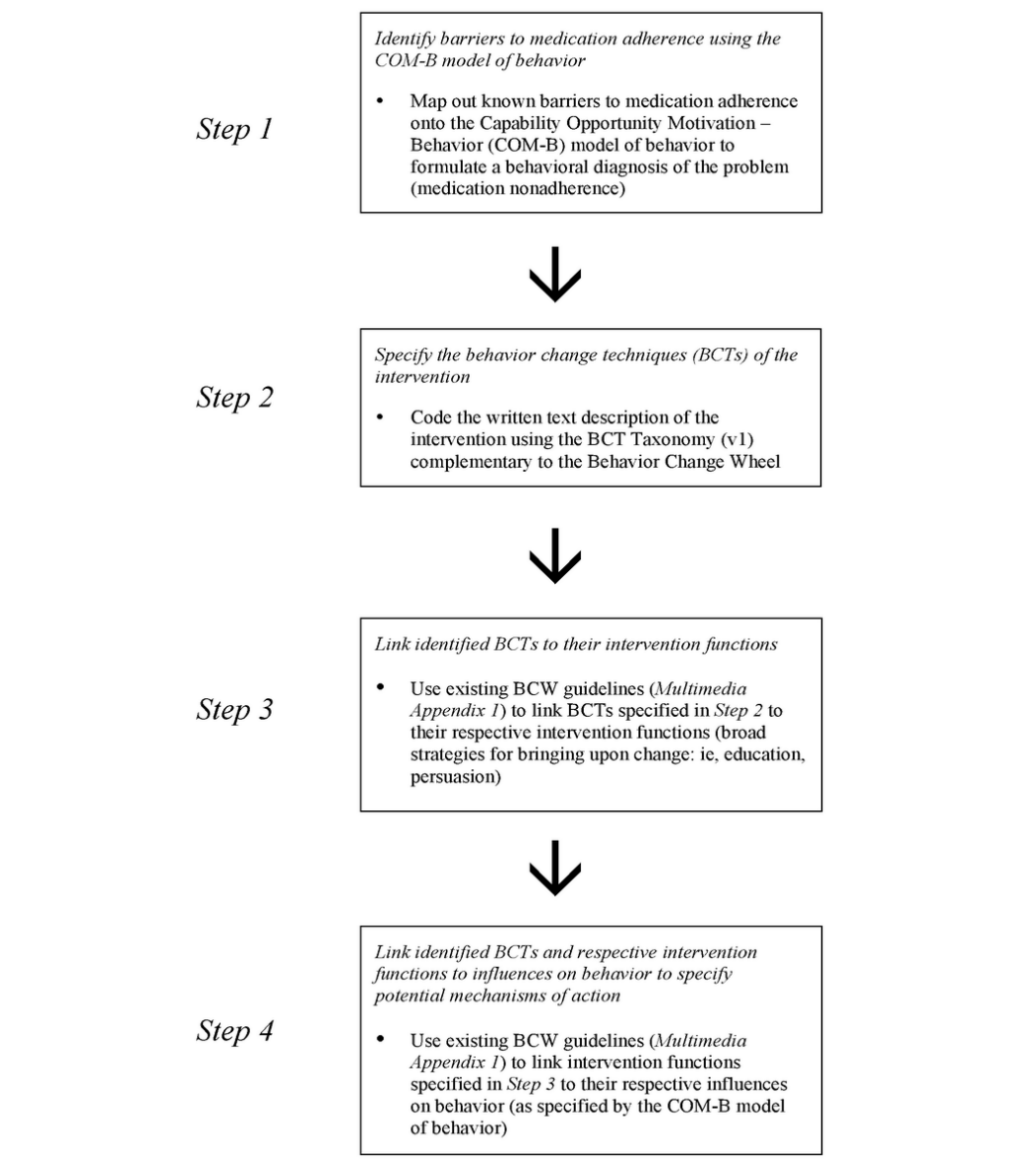
Determining potential mechanisms of action of an intervention using the Behavior Change Wheel (BCW).

In our evaluation of WelTel using the BCW and its complementary taxonomy of BCTs, 4 steps were required to systematically determine the intervention’s potential mechanisms of action for improving medication adherence (Figure 3).

### Step 1: Identify Barriers to Medication Adherence Using the Capability Opportunity Motivation-Behavior (COM-B) Model of Behavior

A *behavioral diagnosis* of ART nonadherence was formulated through the application of the COM-B model of behavior. Known barriers to ART adherence that exist within various countries have been specified in a systematic review [2]. Nine of them considered to be globally relevant were chosen and mapped onto COM-B to reveal influences on behavior that are potentially associated with nonadherence. This process enabled us to gain insight into what needs to change for the target behavior of adherence to occur.

The COM-B model of behavior comprises the core of the BCW, encompassing *capability*, *opportunity*, and *motivation* as the influences on behavior, illustrating the potential interactions between them and the resultant behavior [16]. According to this theory, capability, opportunity, and motivation all need to be present for any behavior to occur, and each component can influence it independently of one another as well as synergistically [18]. Capability is related to one having the psychological (required knowledge and skills) and physical capacity to engage in the behavior. Opportunity is divided into physical (associated with the external environment) and social (ie, cultural norms and interpersonal influences) factors that promote or impede behavior within the individual [16]. Motivation encompasses all brain activity that provokes behavior, including automatic (ie, emotions, wants and needs, and habits) and reflective (ie, belief systems, plans, and evaluations) processes [16].

### Step 2: Specify the Behavior Change Techniques of the Intervention

BCTs delivered by WelTel were characterized using the BCT taxonomy version 1 (BCTTv1). A BCT has been defined as an “observable, replicable, and irreducible component of an intervention designed to change behaviour and a postulated active ingredient within the intervention” [18]. An intervention protocol or manual is typically reviewed to provide the most elaborate description of what it entails. In our evaluation, the WelTel system’s user manual was chosen for analysis, as it provides the most detailed information about the intervention. Furthermore, BCT identification occurred through semistructured discussions within a study team including key stakeholders who have previously collected user feedback from the WelTel platform. During the process of specifying BCTs, all discrepancies were resolved by consensus.

The BCTTv1 serves to characterize an array of behavior change interventions using a standardized language [17]. In the evaluation of established interventions, their delivered content can be specified, synthesized, and linked to the BCW to reveal their intervention functions in order to provide insight into possible active ingredients and mechanisms of action [17]. Upon application, the relationships between the specified BCTs and intervention effectiveness can be investigated; the BCTs that may or may not be effective at changing behavior in addition to other ones not currently being delivered by the intervention have the potential to become identified.

### Step 3: Link Identified Behavior Change Techniques to Their Intervention Functions

Identified BCTs were connected with their respective intervention functions, or broad strategies for bringing upon behavior change, as characterized by the BCW. This process was directed by guidelines provided by the creators of the BCW (Multimedia Appendix 1) that list the BCTs associated with each of the 9 intervention functions.

### Step 4: Link Identified Behavior Change Techniques and Respective Intervention Functions to Influences on Behavior to Specify Potential Mechanisms of Action

To specify potential mechanisms of action, identified BCTs and respective intervention functions were connected to influences on behavior according to the COM-B model (capability, opportunity, and motivation). We followed guidelines provided by the creators of the BCW (Multimedia Appendix 1) that link intervention functions to COM-B influences on behavior; only those thought to be relevant to our study context were included.

## Results

### Step 1: Identify Barriers to Medication Adherence Using the Capability Opportunity Motivation-Behavior (COM-B) Model of Behavior

Formulating a behavioral diagnosis (Multimedia Appendix 2) by mapping out 9 known globally relevant ART adherence barriers onto the COM-B model has revealed that all influences on behavior (physical and psychological capability, physical and social opportunity, and automatic and reflective motivation) may be associated with ART nonadherence, validating the notion that bringing upon this change in behavior is highly complex. The majority of these barriers were linked to more than one COM-B influence on behavior, with reflective motivation and automatic motivation being the most recurring. This implies that both intentional and unintentional forms of nonadherence commonly co-exist within the general adult population. Furthermore, barriers related to a lack of social opportunity (ie, fear of revealing HIV status to family/friends because of stigma and competing priorities) may be appropriately mapped out onto both reflective and automatic motivation. This suggests that facilitating social opportunity could also promote the latter COM-B influences on behavior simultaneously, and this association could even contribute to inducing ART adherence synergistically.

### Step 2: Specify the Behavior Change Techniques of the Intervention

Upon coding the WelTel manual using the BCTTv1 (Multimedia Appendix 3), one BCT was identified for the intervention’s automated text message component and specified as a *prompt/cue*. In contrast, 8 different BCTs were identified for its personalized communication component delivered in response to receiving patient feedback of a problem. These were specified as follows: (1) *prompt/cue*, (2) *unspecified social support*, (3) *reduce negative emotions*, (4) *credible source*, (5) *natural consequences*, (6) *emotional social support*, (7) *practical social support*, and (8) *instruction on how to perform a behavior.*

### Step 3: Link Identified Behavior Change Techniques to Their Intervention Functions

The one BCT identified for WelTel’s automated text message component was linked to one intervention function (*environmental restructuring*; Multimedia Appendix 3). In contrast, the 8 BCTs identified for its personalized communication component were linked to 5 intervention functions (*environmental restructuring, enablement, education, persuasion,* and *training*; Multimedia Appendix 3).

Although the latter component of the intervention appears to be more comprehensive, each of its intervention functions (broad strategies for bringing upon behavior change) can be elaborated upon in terms of several other different BCTs that were not identified in our evaluation (Multimedia Appendix 1). Furthermore, no BCTs were linked to other intervention functions characterized by the BCW (specifically *incentivization, coercion*, and *modeling*). These types of strategies may also be ones to consider given the complexity of ART adherence, as demonstrated by the behavioral diagnosis generated in this study (Multimedia Appendix 2). This suggests that the design of the current intervention could be modified accordingly to potentially have a greater impact on ART adherence.

### Step 4: Link Identified Behavior Change Techniques and Respective Intervention Functions to Influences on Behavior to Specify Potential Mechanisms of Action

On the basis of our evaluation, WelTel’s potential mechanisms of action in promoting ART adherence have been specified as the intervention’s ability to: (1) influence one’s opportunity (both physical and social) and automatic motivation through environmental restructuring and enablement: the delivery of an external trigger, as well as social support, to facilitate adherence behavior; and (2) influence one’s capability (both physical and psychological), opportunity (both physical and social), and motivation (both automatic and reflective) through education, persuasion, training, and enablement: the delivery of health-related information and social support to facilitate adherence behavior.

## Discussion

### Contribution to Behavioral Science Research

In the light of the current literature, this is the first paper to demonstrate how a comprehensive and evidence-based behavior change framework can be applied to an existing two-way mHealth intervention. WelTel has robust evidence for improving ART adherence [19]; therefore, it serves as an optimal means for evaluation using the BCW, an all-encompassing theory that can be appropriately applied worldwide [18]. Our evaluation has systematically revealed its potential mechanisms of action in addressing all influences on behavior regarding ART adherence. This has been specified by the COM-B model of behavior that encourages the critical multilayer examination of the behavior at the individual, interpersonal, and system levels, whereas other established behavioral theories may potentially miss important drivers as not all of these levels of influence are considered.

### One-Way Versus Two-Way mHealth Intervention Strategies: Distinguishing Between Them With Regard to Targeting Nonintentional Nonadherence and Intentional Nonadherence

The application of COM-B highlights the important differences between unintentional and intentional medication nonadherence with regard to their influences on behavior [20,21]. In the context of taking ART daily, unintentional medication nonadherence is often considered simply forgetting to perform the behavior. This factor is related to one’s automatic motivation, described as “processes involving emotional responses, desires (wants and needs), impulses and reflex responses” [18]. In contrast, intentional nonadherence is related to purposeful deviation of the behavior that is associated with reflective motivation, defined as “involving self-conscious planning and evaluations (beliefs about what is good or bad)” [18]. Although they stem from different sources of motivation, both forms of nonadherence are not mutually exclusive from each other, and thus often exist in any one individual prescribed chronic disease treatment (such as ART) over the course of treatment [22,23]. This reality emphasizes the need for adherence-promoting strategies that are multifaceted and target different COM-B influences of behavior.

The distinction between nonintentional and intentional medication adherence behavior is important to consider designing and evaluating interventions. It can provide an explanation as to why interventions solely focused on self-prompting and one-way medication reminders often have failed to show significant impact in clinical trials. This lack of effect has been shown in a randomized control trial using an alarm device for promoting ART adherence [24], in addition to a systematic review evaluating technology-based self-care strategies for improving ART adherence [25]. Moreover, in a systematic review of various mobile technology-based interventions across chronic disease states beyond HIV (ie, diabetes and hypertension), it has been implied that simple text message reminders on their own have modest benefits for influencing adherence [26]. The application of the BCTTv1 for the purpose of evaluating such interventions would likely reveal that the primary BCT being delivered by them is a *prompt/cue* linked appropriately with the intervention function *environmental restructuring*. Connecting this specification with COM-B would indicate that this particular function likely has the potential to only influence one’s physical opportunity (providing a trigger to perform the behavior) and automatic motivation (habit formation through reflex response). Limited effects on other influences on behavior relating to adherence would be expected, including the intervention’s ability to impact one’s reflective motivation, psychological capability, and social opportunity. Therefore, one-way reminder-based interventions may bring upon behavior change with regard to nonintentional nonadherence, but they are unlikely to influence intentional nonadherence. This has been echoed in a meta-analysis comparing one-way versus two-way text message interventions for medication adherence across disease states [9].

Furthermore, it is useful to distinguish between simple two-way text messaging and more complex interactive two-way communication that provides open-ended, enhanced follow-up support tailored to patients’ needs [27]. The former is only one component of the WelTel intervention; however, a recent network meta-analysis that evaluated all types of interventions for HIV adherence globally has classified WelTel as a short messaging service (text message) intervention only [6]. An in-depth analysis of WelTel has revealed a comprehensive blend of mechanisms behind its digital health outreach platform for promoting ART adherence, thus differentiating itself from other mHealth applications considered one-way in nature and other forms of two-way communication that provide a relatively low degree of social support and personalized care. Through consistent weekly automated text messaging and subsequent provider follow-up by phone if patients report a problem or are nonresponsive, WelTel is postulated to deliver a variety of BCTs that comprehensively induces ART adherence. The automated text message component of the intervention is hypothesized to primarily influence one’s physical opportunity and automatic motivation. More importantly, the personalized, interactive patient-provider communication component may have the potential to impact all influences on behavior (physical and psychological capability, social and physical opportunity, and reflective and automatic motivation). Through application of the science of behavior change, we have been able to strengthen the qualitative evidence base for how interactive two-way mHealth interventions may impact both nonintentional and intentional nonadherence.

### Limitations of Our Evaluation Using the Behavior Change Wheel

There were several limitations in using the BCW to evaluate the mHealth intervention. First, coding of an intervention is limited by its documented description (ie, an intervention protocol or manual). Although this method promotes transparency and data validation by external reviewers, information about what the intervention delivers in terms of BCTs may not be captured to its entirety. For example, encouraging *self-monitoring* and *goal setting* could potentially be BCTs delivered independently by the provider upon patient follow-up using the WelTel platform. Evaluating a detailed provider-patient communication log could help determine the presence of such BCTs, and thus contribute to further specifying WelTel’s behavior change mechanisms.

Second, using the BCTTv1 to characterize the mHealth intervention resulted in some ambiguity because of its varied interpretation. In our evaluation, we were uncertain whether or not to code its automated text message component (“Mambo?” or “How are you?”) as a *prompt/cue* and *unspecified social support*, or solely the former. Given WelTel’s personalized communication component for delivering tailored individual care, we decided whatever social support that may be conveyed in the automated text message component would be relatively insignificant. Authors of an mHealth intervention evaluation for smoking cessation have expressed similar sentiments, given the brevity of its text message content and consequently less meaning to draw upon [28]. To address limitations of the taxonomy, one suggestion has been to modify the BCTTv1 for mobile technology–based interventions as opposed to using a generalized classification system for all types of behavioral interventions [26].

Third, behavior change outcomes depend heavily on the patient-provider relationship, which the BCW is unable to account for. Interventions (for medication adherence and retention in care) that prioritize patient confidentiality of HIV status and promote the development of trusting relationships would reduce barriers to their implementation [29,30]. The benefits of personalized care have been echoed in [31] and highlighted in a meta-analysis examining one-way and two-way mHealth interventions for promoting ART adherence [11]. Regular two-way interaction allows for more opportunity to build rapport; therefore, the presence of relatively strong social support (greater enablement) through patient-provider communication by phone would be postulated to enhance the delivery of the BCTs specified in our evaluation.

Overall, the BCW can help to specify what the intervention delivers in terms of content; however, more research is needed to gain a better understanding of other intervention dimensions that play a role in influencing medication adherence (ie, who delivers it, messaging frequency), and how respective processes of change may occur. Another consideration is the behavioral facilitators and barriers that may exist with regard to using the intervention itself. Identifying influences on user engagement with WelTel may elucidate facilitators and barriers at the patient and provider levels, such as comfort level with using the technology and the provider’s style of communication, in addition to knowledge base. It would be wise to identify significant user facilitators and barriers before scaling up the intervention to further optimize its integration into the health care system. The importance of describing user feedback has been emphasized in the mHealth evidence reporting and assessment (mERA) checklist: recent guidelines that have been created to improve the quality of evidence for mHealth through providing a comprehensive and standardized way to report these interventions [32].

### Generalizability of Our Findings

Although WelTel’s digital outreach strategy is thought to address ART adherence more comprehensively than one-way text message engagement platforms, it cannot be assumed that its proposed mechanisms of action would translate across all HIV-positive populations worldwide. A systematic review examining various ART adherence interventions have concluded that behavioral interventions producing a positive impact in one setting may not translate to other populations because of differences in economic, social, and behavioral barriers to adherence [33]. Such barriers including poverty and mental health comorbidities commonly co-exist in various contexts, and addressing these issues through respective integrative services alongside public health HIV interventions may increase implementation success of the latter [34].

Furthermore, it is uncertain if WelTel’s proposed mechanisms of action are generalizable across other chronic disease states. Beyond aiming to promote ART adherence globally and within different populations [13,14,35], WelTel is currently being tested and evaluated for impact on HIV retention in care [36-38], in addition to medication adherence behaviors across other health conditions such as tuberculosis and asthma [39-41]. Arguably, interventions based on a thorough understanding of its target behavior in context will have greater potential for impact. Upon specifying the underlying mechanisms of medication adherence and nonadherence in a given environment, it may be possible to map out the potential value of a WelTel approach. In addition, the mechanisms of action of the intervention may vary by context, and thus the expansion of WelTel to other chronic conditions (as well as target behaviors beyond medication adherence) will require future validation work.

### Implications for Future Research: Our Call to Action

It has been emphasized that replication, implementation, and evidence synthesis are required to gain a more in-depth understanding of behavior change mechanisms and to establish a growing qualitative knowledge base for informing the development of interventions that have greater impact [17]. We hope that our evaluation of the WelTel mHealth service using the BCW is repeated in a similar manner by other researchers involved with mHealth interventions for promoting medication adherence across various disease states and populations. Given the BCW’s established common language of behavior change across interventions, such widespread application of the framework would serve to identify the commonalities and differences between their postulated effects, or lack of. Repeated application would eventually serve to synthesize the current evidence in a systematic manner, and help build a stronger foundation as it relates to specifying how and which combination of BCTs likely have a positive impact, and which ones have minimal influence on medication adherence. With this collective insight, the design of mHealth interventions could be modified accordingly to improve population health outcomes.

### Conclusions

Imposing the BCW onto WelTel has systematically revealed its potential mechanisms of action for improving ART adherence, while deepening our understanding that it may address all influences of this behavior: capability (physical and psychological), opportunity (physical and social), and motivation (reflective and automatic). Our evaluation has revealed that interactive, two-way mHealth interventions may deliver a combination of BCTs that are more likely to capture this to its entirety compared with one-way mHealth interventions, which may only affect automatic motivation and physical opportunity. This has strengthened the evidence base for how and why a two-way design may be more effective than a one-way design at addressing both nonintentional and intentional forms of nonadherence. The application of the BCW for evidence synthesis across mHealth interventions targeting various chronic diseases would contribute to strengthening the knowledge base regarding how they may work to impact medication adherence behavior.

## Appendix

Multimedia Appendix 1 Intervention functions: linkages with BCTs and COM-B.

Multimedia Appendix 2 Behavioral diagnosis of ART nonadherence.

Multimedia Appendix 3 Evaluating WelTel: characterizing its delivered BCTs, linking them to their intervention functions and COM-B.

## Abbreviations

ART: antiretroviral therapy
BCW: Behavior Change Wheel
BCT: behavior change technique
BCTTv1: behavior change technique taxonomy (version 1)
COM-B: Capability Opportunity Motivation-Behavior
