# Supplementary material for: Interactive Two-Way mHealth Interventions for Improving Medication Adherence: An Evaluation Using The Behaviour Change Wheel Framework
Source: JMIR Mhealth Uhealth. 2018 Apr 12;6(4):e87. doi: 10.2196/mhealth.9187 (PMC5920150; doi:10.2196/mhealth.9187)
Supplement: Multimedia Appendix 1 [file mhealth_v6i4e87_app1.pdf]

Appendix 1: Intervention functions: linkages with BCTs and COM-B
[a]As described in [18]

Intervention function and definition[a]	Frequently used BCTs associated with intervention function[a]	How intervention functions potentially influence behavior through COM-B[a]
Education<br><br>"Increase knowledge or understanding"	"Information about social and environmental consequences"<br>"Information about health consequences"<br>"Feedback on behavior"<br>"Feedback on outcome(s) of behavior"<br>"Prompts/cues"<br>"Self-monitoring of behavior"	Psychological capability<br><br>Reflective motivation
Persuasion<br><br>"Using communication to induce positive or negative feelings or stimulate action"	"Credible source"<br>"Information about social and environmental consequences"<br>"Information about health consequences"<br>"Feedback on behavior"<br>"Feedback on outcome(s) of the behavior"	Automatic motivation<br><br>Reflective motivation
Incentivization<br><br>"Creating an expectation of reward"<br><br>*Coercion*<br><br>"Creating an expectation of punishment or cost"	"Feedback on behavior"<br>"Feedback on outcome(s) of behavior"<br>"Monitoring of behavior by others without evidence of feedback"<br>"Monitoring outcome of behavior by others without evidence of feedback"<br>"Self-monitoring of behavior"	Automatic motivation<br><br>Reflective motivation
Restriction<br><br>"Using rules to reduce the opportunity to engage in the target behavior"	No linked BCTs specified	Social opportunity<br><br>Physical opportunity

Appendix 1 (continued): Intervention functions: linkages with BCTs and COM-B
[a]As described in [18]

Intervention function and definition[a]	Frequently used BCTs associated with intervention function[a]	How intervention functions potentially influence behavior through COM-B[a]
Enablement<br><br>"Increasing means or reducing barriers to increase capability (beyond education and training) or opportunity (beyond environmental restructuring)"	"Social support (unspecified)"<br>"Social support (practical)"<br>"Goal setting (behavior)"<br>"Goal setting (outcome)"<br>"Adding objects to the environment"<br>"Problem solving"<br>"Action planning"<br>"Self-monitoring of behavior"<br>"Restructuring the physical environment"<br>"Review behavioral goal(s)"<br>"Review outcome goal(s)"	Physical capability<br><br>Psychological capability<br><br>Physical opportunity<br><br>Social opportunity<br><br>Automatic motivation
Training<br><br>"Imparting skills"	"Demonstration of behavior"<br>"Instruction of how to perform a behavior"<br>"Feedback on the behavior"<br>"Feedback on outcome(s) of behavior"<br>"Self-monitoring of behavior"<br>"Behavioral practice/rehearsal"	Physical capability<br><br>Psychological capability<br><br>Physical opportunity<br><br>Automatic motivation
Environmental restructuring<br><br>"Changing the physical or social context"	"Adding objects to the environment"<br>"Prompts/cues"<br>"Restructuring the physical environment"	Physical opportunity<br><br>Social opportunity<br><br>Automatic motivation
Modeling<br><br>"Providing an example for people to aspire to or imitate"	"Demonstration of the behavior"	Social opportunity<br><br>Automatic motivation
