# Supplementary material for: Interactive Two-Way mHealth Interventions for Improving Medication Adherence: An Evaluation Using The Behaviour Change Wheel Framework
Source: JMIR Mhealth Uhealth. 2018 Apr 12;6(4):e87. doi: 10.2196/mhealth.9187 (PMC5920150; doi:10.2196/mhealth.9187)
Supplement: Multimedia Appendix 2 [file mhealth_v6i4e87_app2.pdf]

Multimedia Appendix 2: Behavioral diagnosis of ART non-adherence<br>[a]COM-B model of behavior definitions in [18]						
Barriers to ART adherence globally [2]	*BEHAVIORAL DIAGNOSIS (DETERMINING WHAT NEEDS TO CHANGE):* KNOWN BARRIERS TO ART ADHERENCE LINKED WITH POTENTIAL INFLUENCES OR MECHANISMS FOR INHIBITING THIS BEHAVIOR					
	CAPABILITY[a]		*OPPORTUNITY*[a]		*MOTIVATION*[a]	
	Physical	*Psychological*	*Physical*	*Social*	*Reflective*	*Automatic*
	"Having the physical skills, strength or stamina to perform the behavior"	"Having the knowledge, psychological skills, strength or stamina to perform the behavior"	"What the environment allows or facilitates in terms of time, triggers, resources, locations, physical barriers, etc."	"Including interpersonal influences, social cues and cultural norms that influence our way of thinking"	"Processes involving intentional planning and evaluation (beliefs about what is good or bad)"	"Processes involving emotional reactions, desires (wants and needs), inhibitions and reflex responses"
Fear of revealing HIV status to family/friends due to stigma				✓	✓	✓
Simply forgetting to take medication						✓
Treatment skepticism (doubts about efficacy and long-term effects)		✓			✓	✓
Unclear on the importance of daily adherence		✓				
Feeling healthy and fine, believing that treatment is unnecessary		✓			✓	
Medication access (financial and transportation difficulties to receive treatment and health care services)			✓			
Competing priorities (i.e. work and family responsibilities), too busy to properly comply			✓	✓	✓	✓
Difficulties in understanding treatment instructions		✓				
Real/anticipated side effects	✓				✓	✓
