# Supplementary material for: Interactive Two-Way mHealth Interventions for Improving Medication Adherence: An Evaluation Using The Behaviour Change Wheel Framework
Source: JMIR Mhealth Uhealth. 2018 Apr 12;6(4):e87. doi: 10.2196/mhealth.9187 (PMC5920150; doi:10.2196/mhealth.9187)
Supplement: Multimedia Appendix 3 [file mhealth_v6i4e87_app3.pdf]

Multimedia Appendix 3: Evaluating WelTel: characterizing its delivered BCTs, linking them to their intervention functions and COM-B
[a]BCT definitions from [18]. Refer to *Multimedia Appendix 2* for COM-B model definitions. Linkages specified in *Multimedia Appendix 1*.

(1) Automated text message component<br>*(2)(3)(4) Personalized communication component*		CAPABILITY		OPPORTUNITY		MOTIVATION	
Text description of intervention (from WelTel user manual)	*Identified BCT*[a]<br>*Intervention Function* (refer to *Multimedia Appendix 1* for individual definitions)	PHYSICAL	PSYCH	PHYSICAL	SOCIAL	REFLECT	AUTOMATIC
(1) "Patients received weekly message saying 'Mambo' (how are you?), must reply 'Sawa' (fine) or 'Shida' (not fine)"	*7.1 (prompts/cues):* "Introduce or define environmental or social stimulus with the purpose of prompting or cueing the behavior"<br>*Environmental restructuring*			✓			✓
(2) "The clinician then called patients who said they had a problem or who failed to respond within 2 days."	*3.1 (social support - unspecified):* "Advise on, arrange or provide social support or non-contingent praise or reward for performance of the behavior. It includes encouragement and counselling, but only when it is directed at the behavior"<br>*7.1 (prompts/cues):* "Introduce or define environmental or social stimulus with the purpose of prompting or cueing the behavior"<br>*Enablement, Environmental restructuring*			✓	✓		✓
(3) "The clinician appropriately provides patient with personalized support, such as addressing stigma around HIV/AIDS, provision of education relating to the importance of adhering to treatment, and the consequences of not engaging in this behavior"	*11.2 (reduce negative emotions):* "Advise on ways of reducing negative emotions to facilitate performance of the behavior<br>*9.1 (credible source):* "Present verbal or visual communication from a credible source in favour of or against the behavior"<br>*5.1 (natural consequences):* "Information about health consequences"<br>*3.3 (social support - emotional):* "Advise on, arrange, or provide emotional support for performance of the behavior"<br>*Education, Persuasion, Enablement*		✓	✓	✓	✓	✓
(4) "The clinician may help to clarify patient ART dosing regimen upon confusion, as well as how to manage adverse effects"	*3.2 (social support - practical):* "Advise on, arrange, or provide practical help for performance of the behavior"<br>*4.1 (instruction on how to perform a behavior):* "Advise or agree on how to perform the behavior"<br>*Training, Enablement*	✓	✓	✓	✓		✓
